# The effect of maternal nicotine on basement membrane collagen IV of brain microvessels changes in neonatal Balb/C mice

**Published:** 2014-04

**Authors:** Somayyeh Sadat Tahajjodi, Maryam Amerion, Nasser Mahdavi Shahri, Mehdi Jalali, Mohammad Reza Nikravesh

**Affiliations:** 1 *Department of Biology, Faculty of Sciences, Ferdowsi University of Mashhad, Mashhad, Iran.*; 2 *Department of Anatomy, School of Medicine, Mashhad University of Medical Sciences, Mashhad, Iran.*

**Keywords:** *Nicotine*, *Basement membrane*, *Collagen type IV*, *Brain microvessels*

## Abstract

**Background:** Nicotine can pass through placental blood barrier and accumulate in the developing organs of fetus. Also, entering the breast milk, nicotine can have an effect on the neonates. Investigations have showed that collagen IV is one of the most important micro vessels basement membrane components.

**Objective:** In this study, the effect of maternal nicotine exposure in pre and postnatal periods on collagen IV in microvessels of neonatal Balb/C mice brain cortex was studied by immunohistochemistry technique.

**Materials and Methods:** 24 pregnant Balb/C mice were divided in to 4 groups (6 mice in each group): two experimental and 2 control groups. The mothers in the 1^st^ experimental group were injected 3 mg/kg nicotine intrapritoneally from the 5^th^ day of pregnancy to parturition daily and in 2^nd^ experimental group the same procedure was repeated to the 10^th^ day after parturition (lactation). The control groups received the same volume of normal saline during the same time. 10 days after delivery, the brain tissues of newborns were isolated. Then, prepared blocks from fixed brain were cut serially for immunohistochemical assay.

**Results: **The findings of the present study indicated that collagen IV reaction in microvessels basement membrane in the first experimental group increased significantly compared to the first control group (p=0.002). In addition, collagen IV reaction in microvessels basement membrane in the 2^nd^ experimental group increased significantly compared to the 2^nd^ control group (p=0.002). However, no significant difference was observed between the two experimental groups.

**Conclusion:** These results suggested that maternal nicotine exposure during prenatal period may increase basement membrane collagen IV expression. Also, nicotine increases in maternal breast milk has no effect on basement membrane collagen IV expression.

## Introduction

Nicotine as a plant alkaloid and main component of cigarette has many toxic and teratogenic effects on the body. These effects have been proven by many studies. Nicotine can pass through placental blood barrier easily and accumulate in amniotic fluid as much as 15% more than maternal serum, inducing negative effects on developing organs during fetal period (1). Many studies have demonstrated that nicotine exposure during pregnancy can have negative effects such as: intrauterine growth retardation (IUGR), birth weight reduction, and physical behavioural disorders (1-3). Nicotine is related with increased premature parturition, the reduction of placental blood flow sudden abortion and sudden infant death syndrome (SIDS) (2, 3). The cardiovascular effects of nicotine results in reduced blood flow to the placenta (4). Vascular development needs the correct interactions among the endothelial cells, the surrounding cells and the matrix. These interactions include many cell adhesion interactions, such as cell-matrix interactions both with basement membranes, and with the surrounding extracellular matrix (5). Endothelial cells produce and bind to multiple basement membrane components (6). Some molecules such as collagen IV, laminin, fibronectin, and heparin sulfate form basement membrane structure (7). Fibronectin and collagens seem to promote migration and proliferation, whereas basement membrane collagen IV and laminin stimulate attachment and differentiation (6). The formation of collagen IV during angiogenesis process is one of the most important events in fetus development (7).

Nicotine has direct negative effects on the cardiovascular system. Several studies have shown that many of cardiovascular system illnesses are more common in smokers than nonsmokers (8-11). The chronic exposure to nicotine has been observed to play a pathogenic role in the induction and progression of cardiovascular disorders like cardiomyopathy and peripheral vascular diseases (10-11). Nicotine alters the function of vascular endothelium and may affected basement membrane (12). Also, it has been shown that nicotine alters gene expression in endothelial cells (13). Nicotine effects on arteriolar fibrosis, myocardial fibrosis and stiffness and oral fibrosis by increasing the expression of all types of collagens (14-16).

According to these studies, it is speculated that the direct effects of nicotine on vessels structure and basement membrane may be predictable. The purpose of this study was to examine the direct effect of maternal nicotine exposure on collagen IV basement membrane of brain microvessels during pregnancy and lactation.

## Materials and methods

This study is an experimental and basic study and all ethical rules were considered about the mice. The subjects used in the study were 24 Balb/c mice obtained from Mashhad University of Medical Sciences. The environmental conditions were equal for all (23-25^o^C, relative humidity 50-55%, 12 hr light-dark cycle, light on at 6.00 am). After mating, these 24 mice were randomly assigned to two experimental and two control groups. The mothers in the first experimental group were injected 3 mg/kg nicotine (Serva Fein Biochemical company Germany) dissolved in normal saline intrapritoneally from the 5^th^ day of pregnancy until parturition on a daily basis. However, those in the second experimental group had the same amount of nicotine injection until the 10^th^ day after parturition. The mice in the two control groups received the same amount of normal saline instead of nicotine from the 5^th^ day of pregnancy until the parturition and 10 days after parturition respectively. 

Ten days after delivery, the mothers were separated from their newborns (176 newborns in 4 groups). Then, the newborns were anesthetized by ether and were perfused transcardially with paraformaldehyde 4%. Their brain tissues were isolated and fixed for 48 hours at room temperature. After fixation, the tissues were dehydrated by passing through a series of solutions of increasing Isopropyl alcohol (IPA). Then the tissues were infiltered with paraffin and finally embedded within the small cube of paraffin. The prepared blocks were cut serially in 5-microne coronal sections. One out of each ten sections was selected from the brain tissues. After deparaffination and rehydration, the selected sections were placed in Triton X-100 for 10 minutes and then incubated with monoclonal anti collagen IV for 2 hours (anti mouse anti rabbit, Dako Cytomtaion company, Denmark). After being washed with PBS, they were placed for 10 minutes in Di-aminobenzidine, and again washed for 10 minutes with PBS. Finally, they were stained with hematoxylin and mounted with enthelan (17-18). 

Based on the collagen IV expression levels in different part of brain tissue, sections will show positive coloring reaction to the used anti body, from light to dark brown. Because the rate of coloring reaction is the determinant of collagen IV density, Gong method (Color intensity grading by three separate observers) was used to grad the coloring reaction from zero to 4+ (19). A microscope camera was used to take photographs. Then, two different individuals were asked to grade the colorings in the photographs. 

**Statistical analysis**

Statistical analyses were done by SPSS software version 16 using nonparametric Kruskal Wallis and Mann-Withney U tests for collagen IV reaction (p<0.01).

## Results

**Figure 1:**
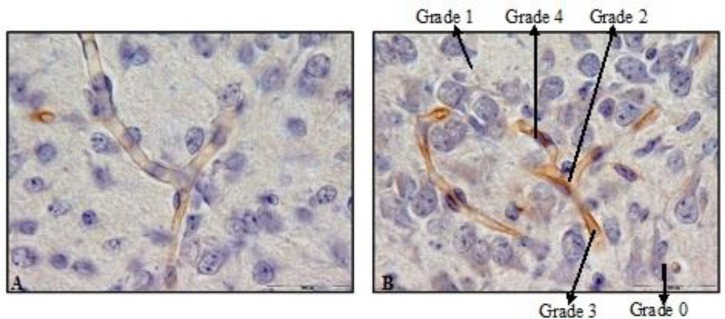
Immunohistochemistry staining by anti-collagen IV antibody in 10 days old newborns brain cortex of control 1 (A) and experimental 1 (B) groups (magnification 100X). A significant difference was observed in collagen IV reaction in microvessels basement membrane between control 1 and experimental 1 groups. Grading is based on Gong method

The data analysis showed that the intensity of coloring reaction in the first experimental group was significantly higher than that of the control (p<0.01). Therefore, the collagen IV expression was significantly different in the 1^st^ experimental group and the 1^st^ control group (p=0.002) (Figure 1, Table I). Similarly, the coloring reactions of the second experimental and control groups were significantly different (p=0.002). 

**Figure 2:**
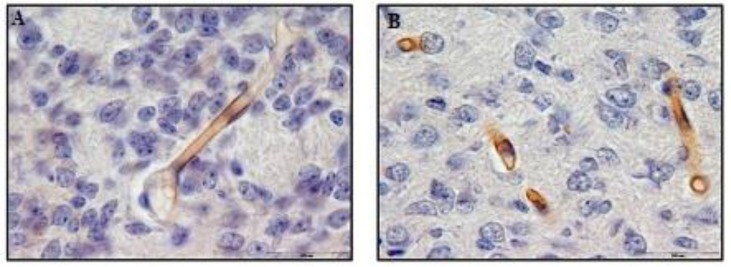
Immunohistochemistry staining by anti-collagen IV antibody in 10 days old newborns brain cortex of control 2 (A) and experimental 2 (B) groups (magnification 100X). A significant difference was observed in collagen IV reaction in microvessels basement membrane between control 2 and experimental 2 groups

**Figure 3:**
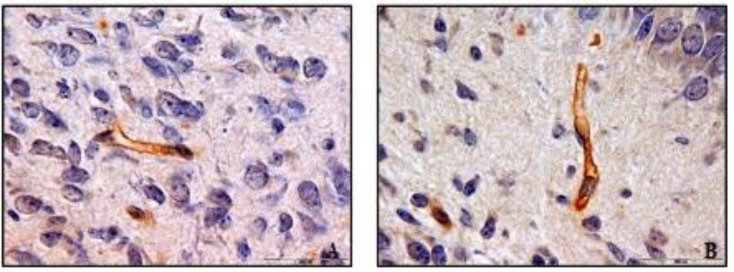
Immunohistochemistry staining by anti-collagen IV antibody in 10 days old newborns brain cortex of experimental 1 (A) and experimental 2 (B) groups (magnification 100X). No significant difference was observed in collagen IV reaction in microvessels basement membrane between experimental 1 and experimental 2 groups

This difference in coloring reaction was because of the difference in the collagen IV expression in the experimental and control groups (Figure 2 Table I). However, the analysis of the data showed that the collagen IV expression was not significantly different (p=1) in the first and second experimental groups although the nicotine administration was different for them (Figure 3).

**Table I:** Comparison between Collagen IV reactivity in basement membrane (BM) in the experimental and control groups

**Variability**	**Experimental 1**	**Control 1**	**Experimental 2**	**Control 2**	**p- value**
Collagen IV reactivity in BM	3 (1-4)	1 (0-3)			p<0.01
Collagen IV reactivity in BM			3 (1-4)	1 (0-3)	p<0.01

Data are shown as median (min- max)

## Discussion

Cardiovascular system is widely affected by nicotine. Several studies have showed that many of cardiovascular system illnesses are more common in smokers than nonsmokers (8-11). Also, nicotine effect on vascular pathology should not be ignored (20). Vascular development requires correct interactions among endothelial cells, surrounding cells and matrix (5). Because basement membrane is a part of extracellular matrix, it has deep effect on formation and function of vascular endothelium, such as progress in migration, proliferation, adherence and differentiation. Basement membrane role is considerable in vascular pathology (6). So, non-formation or incomplete formation of basement membrane leads to disorders in vascular system (21).

The studies of nicotine effect on collagen in various organs have had different results. The review of these studies has shown that nicotine has effects on arteriolar fibrosis, myocardial fibrosis and stiffness and oral fibrosis by increasing the expression of all types of collagens (14-16). Smoking has a negative effect on wound and tissue healing. Smoking affects collagen synthesis and deposition of collagen in the wound .The increasing of collagen degradation probably is cause the reduction in fibroblast migration and proliferation and enhance in neutrophil collagenase (MMP-8) released from inflammatory cells. Also, smoking caused depresses tissue oxygenation and low vitamin C levels. These effects probably interfere with the molecular pathways of collagen metabolism (22). In other studies, mRNA level of type II collagen was up regulated by nicotine in chondrocytes (23, 24).

On the other hand, many studies on skin and bone have shown that nicotine can cause reduction in expression of collagen types (25, 26). Also, a study on nicotine effect on collagen expression in wound scar tissue did not show any difference in expression of genes related to this protein (27). In present study, results indicated that nicotine had a significant positive effect on collagen IV expression; similar nicotine-induced stimulation was noted by other mentioned study. Based on these findings, it is suggested that the increase in collagen IV expression in experimental groups is the result of being exposed nicotine during fetal period.

Because of its features such as low molecular weight and solubility in water, nicotine can pass through placental blood barrier easily and affect fetal developing organs (1). So, maternal nicotine exposure can affect the amount of collagen in various developing organs of the fetus. There are some studies investigating this. In some studies maternal nicotine effect on collagen genes expression in respiratory connective tissue and vessels basement membrane, bronchiole basement membrane, lung extracellular matrix, trophoblastic and chorionic villi has been studied (18, 28-30). In all these studies, nicotine caused an increase in collagen type’s expression, all of which are consistent with the obtained results in the present study.

In these studies, an increase in collagen amount was attributed to the increase in collagen genes expression mRNA (28). In addition, the increase in CCN2 amount (connective tissue growth factor) caused by stimulation with nicotine was introduced as the cause of increasing amount of collagen (16). In another study, it was suggested that nicotine might interact with α7 nicotinic acetylcholine receptors (α7nAchR) directly and cause accumulation of collagen in tissue (28). In the present study mice neonatal brain micro vessels basement membrane was examined using maternal nicotine. These results, show that maternal nicotine can increase collagen IV in brain micro vessels basement membrane probably by up regulating collagen IV genes expression mRNA.

On the other hand, many studies have shown that being exposed to nicotine during lactation can cause accumulation of nicotine in mammary gland and affect newborn through nicotine entrance to body with breast milk. Milk containing nicotine is absorbed through the gastrointestinal tract and accumulates in some tissues (31). Many studies focused on the negative effects of nicotine on milk quantity during lactation because of a decrease in prolactin hormone and the tendency to breastfeeding (32). In this study, two experimental groups were compared in order to investigate the effects of nicotine on micro vessels basement membrane collagen IV during lactation. The results of this study suggest that there is no significant difference between two experimental groups. This result may show that being exposed to nicotine during lactation cannot cause change in collagen IV expression. 

## Conclusion

The overall conclusion is that maternal nicotine may cause an increase in collagen IV expression in micro vessels basement membrane. This may lead to a change in basement membrane thickness thus influencing the functioning of this structure. Further studies on the molecular mechanism would be necessary to perform to bring to light how maternal nicotine and smoking during pregnancy affect vascular and basement membrane structure and disorders.

## Conflict of interest

There is no conflict of interest.

## Footenote

***This article extracted from M.Sc. thesis. (Somayyeh Sadat Tahajjodi))***
